# Dementia, the Life Course and Health Outcome Preferences

**DOI:** 10.1192/bjo.2026.11166

**Published:** 2026-06-30

**Authors:** Thomas McConnell, Jo Coast

**Affiliations:** 1 Lancashire and South Cumbria Foundation Trust, Preston, United Kingdom; 2 University of Bristol, Bristol, United Kingdom

## Abstract

**Aims:**

The global burden of dementia is escalating rapidly, with prevalence estimates rising from 57.4 million in 2019 to a projected 152.8 million by 2050. This increase carries profound economic implications; direct and indirect costs are forecasted to consume approximately 0.5% of global GDP by 2050. Health economic evaluation can support efficient allocation of resources but key challenges remain in determining what outcomes are valued and how. We discuss critically the role of the life course approach in informing health economic evaluations.

**Methods:**

Various methods exist for determining the relative costs and benefits of different treatment approaches. Relatively simple methods use pre-determined quality of life values for different stages of dementia to model the ‘utilities’ generated by treatment. The more recent ICECAP approach includes a broader range of outcomes than just health states by considering aspects of capability.

**Results:**

Despite these innovations in determining health outcome measures challenges remain. Models must account for complex interactions between symptoms, disease progression and quality of life. Another challenge is accounting for the “Proxy-Patient” gap, where caregiver reports often diverge from patient experiences. Furthermore,economic models can struggle with the valuation of “process utility” and the difficulties of extrapolating data from brief clinical studies into long-term disease models spanning many years.

The Life Course Approach:Our work proposes integrating a ‘life course’ perspective into health economic approaches to better understand dementia within the context of a patient’s entire life. By utilizing the concepts of “Trajectories and Transitions”,economists can evaluate how dementia disrupts expected life paths–such as the loss of a planned retirement or the inability to fulfil the role of a grandparent. Additionally, the concept of “Linked Lives” offers insight into the social interdependencies that shape the “Proxy-Patient” gap. Qualitive methods offer an important lens to investigate these problems.

**Conclusion:**

Incorporating life course and qualitative approaches could provide a more robust framework for valuing preferences in dementia. By capturing the impact of disrupted life expectations and social interconnectedness, economic analyses can more accurately reflect the true impact of the disease, leading to more equitable and efficient resource distribution.

